# Predictive value of EEG-derived pain threshold index for acute postoperative pain in children

**DOI:** 10.3389/fped.2022.1052532

**Published:** 2022-12-21

**Authors:** Jingjing Lv, Jianwei Zhang, Kan Zhang, Jijian Zheng

**Affiliations:** ^1^ Department of Anesthesiology, Shanghai Children's Medical Center, School of Medicine, Shanghai Jiao Tong University, Shanghai, China; ^2^ Center for Brain Science, Shanghai Children's Medical Center, Shanghai Jiao Tong University School of Medicine, Shanghai, China

**Keywords:** emergence agitation, postoperative pain, pain threshold index, surgical pleth index, children

## Abstract

**Background:**

Electroencephalogram (EEG)-derived pain threshold index (PTI) has been developed as a novel pain recognition indicator and has been proved to be useful in the prediction of acute postoperative pain in adults. Evidence of its usability in children is limited. The aim of this study was to investigate the prediction value of this novel pain indicator PTI for acute postoperative pain in children.

**Methods:**

A total of 80 patients undergoing laparoscopic surgery under general anesthesia were enrolled. Blood pressure, heart rate (HR), surgical pleth index (SPI), PTI, and EEG-derived sedative index-wavelet index (WLI) data were recorded at the end of the surgery. The postoperative pain scores Face, Legs, Activity, Cry, Consolability (FLACC) were obtained in the emergence room 5 min after the children wake up. Receiver-operating characteristic curve was performed to analyze the predictive value of PTI, SPI, HR, and mean arterial pressure (MAP). The consistency between SPI and PTI was also evaluated.

**Results:**

Results showed that the areas under curves (95%CI) of PTI and SPI were 0.796 (95% CI: 0.694–0.895) and 0.753 (95% CI: 0.632–0.874), respectively, with the best cut-off value of 58 and 45 to discriminate between mild and moderate to severe pain.

**Conclusion:**

This study suggested that PTI obtained at the end of the surgery could predict acute postoperative pain in children with an acceptable accuracy. It will help with early recognition and treatment of postoperative pain, thus reducing the pain in children. In addition, PTI had a good consistency with SPI in predicting acute postoperative pain in children.

## Introduction

Emergence agitation (EA) is the most common adverse event during early recovering from general anesthesia, especially for preschool-aged children, presenting as excitation, restlessness, nonpurposeful movement, inconsolability, and disorientation, which significantly increases the risk of self-injury and prolonged hospital stay and requires additional nursing staff and medical care costs (1, 2). However, it is hard to distinguish pain-related screaming, irritability, etc., symptoms from those of EA due to lack of effective and objective pain assessment methods.

Timely identification and management of postoperative pain would be helpful to reduce or prevent EA incidence. However, it is very challenging to reach an appreciable analgesic level without respiratory depression due to the lack of reliable pain assessment tools, especially for those pediatric patients during early recovery from general anesthesia. Many objective pain assessment methods, such as pupil diameter, skin conductance (SC), heart rate variability (HRV), analgesia/nociception index (ANI), and surgical pleth index (SPI), have been used to evaluate the intensity of noxious stimulation and guide intraoperative analgesia under general anesthesia in adult patients (3–6). However, few of them are used in children and to predict postoperative pain.

Recently, a novel pain recognition index, pain threshold index (PTI) based on integrated electroencephalogram (EEG) wavelet analysis was developed to reflect the antinociceptive state under general anesthesia by Beijing Easymonitor Technology Co., Ltd, Beijing, China. The theoretical base of the PTI algorithm was based on dissecting the scalp EEG signals into both cortical and subcortical signals by EEG wavelet analysis. Our previous study showed that PTI can be used to predict the hemodynamic reactivity induced by endotracheal intubation and skin incision in pediatric patients (7). Additionally, PTI measured at the end of surgery can predict the occurrence of acute postoperative pain in adult patients, and a good agreement between SPI and PTI values exists at the end of surgery (8). Whether PTI can be used to predict postoperative pain in children is unknown. Our primary aim was to explore whether the PTI can be used to predict postoperative pain in children aged 2–7 years. Our secondary aim was to compare the predictive performance of postoperative pain in children between PTI and SPI, an easily acquired and well-studied parameter of intraoperative nociception in adult.

## Materials and methods

**Figure 1:**
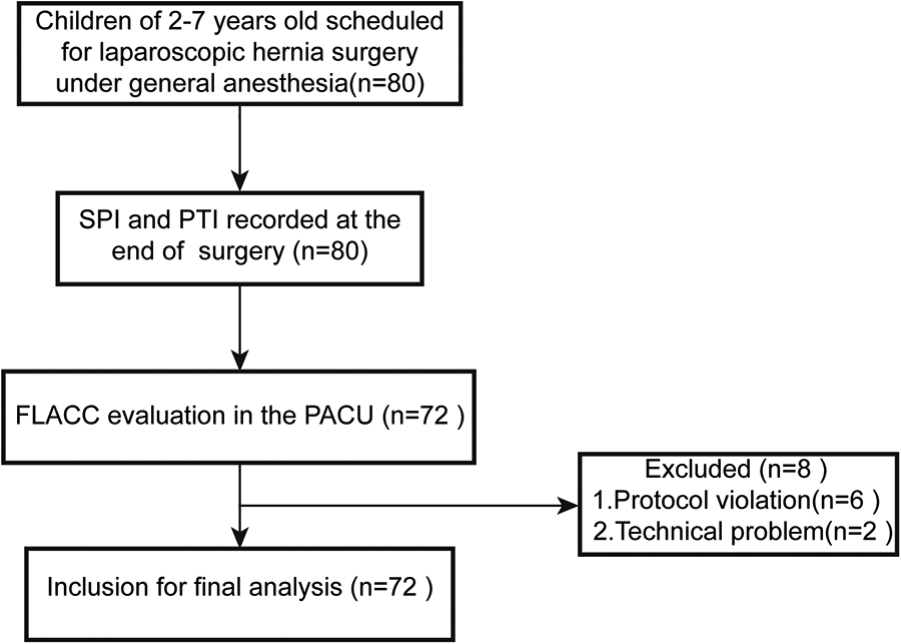
Flow diagram of the study protocol. SPI, surgical pleth index; PTI, pain threshold index; PACU, post-anesthesia care unit.

This prospective observational clinical study was approved by the Ethics Committee of the Shanghai Children's Medical Center (SCMCIRB-K2021003-1) and registered on the Chinese Clinical Trials Registry (ChiCTR2100054032). The study was performed between December 10, 2021, and August 30, 2022. After obtaining written informed consents from parents, the patients undergoing elective surgery of laparoscopic hernia repair were enrolled in this prospective observational study. The flow diagram of this trial is shown in Figure 1.

### Patients

The pediatric patients of American Society of Anaesthesiologists (ASA) I–II aged 2–7 years who were scheduled for nonemergency laparoscopic hernia surgery under general anesthesia were included. Exclusion criteria included preoperative chronic pain history, neuropsychiatric disease, craniotomy history, severe cardiovascular disease, drug dependence, arrhythmia and pacemaker implantation history, use of vasoactive drugs during data collection and intraoperative use of ketamine, *β* receptor blockers, or any other drugs suspected of interacting with sympathetic nerve balance.

### Anesthetic technique

For pediatric patients younger than 4 years old, 0.5 mg/kg midazolam was administered orally as premedication 20–30 min before entering the operating room. Other patients without premedication received 0.1 mg/kg midazolam IV during induction. The standard anesthetic management was adopted during perioperative period. In brief, all the patients were induced with propofol (2–4 mg/kg), fentanyl (2 µg/kg), and rocuronium (0.6 mg/kg). After successful intubation, ultrasound-guided bilateral abdominal transverse fascia block was conducted before surgery. General anesthesia was maintained with propofol (5 mg/kg h) and remifentanil (0.2–0.3 µg/kg min), with or without sevoflurane according to the hemodynamic changes during the surgery. In addition to standard monitoring, data on blood pressure (BP), heart rate (HR), SPI, EEG-derived sedative index-wavelet index (WLI), and PTI were recorded at the end of the surgery (during skin closure). During the data collection period, the WLI was not permitted to increase to >60. To avoid interference from the medications, vasoactive drugs and naloxone were not used. Patients will be excluded from this study if these drugs are necessary. The patients were sent to the post-anesthesia care unit (PACU) after extubation for recovery. The Face, Legs, Activity, Cry, Consolability (FLACC) evaluation was performed 5 min after the patients woke up. The children were considered awake when they were able to open their eyes when called and can respond purposefully. If the pain score was greater than 4 points, then pain treatment was guided by our standard protocol in PACU and not influenced by this study.

### SPI and PTI data collection and calculation

SPI measurement was performed from a finger on the side opposite to the Non-Invasive Blood Pressure (NIBP) measurement through a saturation sensor (GE Healthcare, Helsinki, Finland). The plethysmographic waveform was continuously recorded and reanalyzed to produce the normalized pulse plethysmographic amplitude (PPGAnorm), and the normalized heartbeat interval (HBInorm). The published equation for the value (0–100) is SPI = 100 − (0.7 × PPGAnorm + 0.3 × HBInorm) (9).

EEG-derived WLI and PTI were monitored with a multifunction monitor (Beijing Easymonitor Technology Co., Ltd., China). After the skin of the forehead and mastoid was cleaned with alcohol cotton pads, the EEG collection electrodes were placed on the patients’ forehead and mastoid. The placement of the electrodes was followed by the instruction from a previous study (7). Real-time EEG signals were collected for WLI and PTI calculation, and the data were displayed on the monitor screen in pediatric mode.

The principle of the EEG-derived PTI calculation is based on the wavelet algorithm, which was described in detail in a previous study (7). Briefly, the EEG data were recorded using the HXD-I monitor, and EEG signal processing was demonstrated by complex algorithm formulas and calculation methods through EEG analysis software package developed by Beijing Easymonitor Technology Co., Ltd. Then, it was preprocessed *via* the continuous wavelet transforms, binary discrete wavelet transforms, and the frequency domain reconstruction algorithm. Furthermore, it was analyzed by the waveform recognition algorithm, the spectral analysis algorithm, and the wavelet analysis algorithm. Finally, the values of WLI (0–100) and PTI (0–100) are displayed on the monitor screen in real time as dimensionless scores, and the values are updated every 2 s.

### Statistical analysis

Statistical analyses were performed using the IBM SPSS Statistical 21.0 (IBM Corp., Armonk, NY, United States) and GraphPad Prism 8.0.1 (GraphPad Inc., United States), and *P* < 0.05 was considered as statistically significant. According to the results of previous research, area under the curve (AUC) of PTI was 0.772. We assume an AUC of 0.75 with *α* = 0.05, *β* = 0.10, and a 15%–20% loss of patients because of protocol violations; the sample size calculated was 66. We finally recruited 80 patients and 72 were included for final analysis. Continuous data were expressed as mean ± standard deviations (SD) while categorical variables were presented as category counts. Bland–Altman analysis was used to characterize the consistency between SPI and PTI. Receiver-operating characteristic (ROC) curves and the associated AUC were analyzed to characterize the predictive value of SPI, PTI, HR, and mean arterial pressure (MAP) for postoperative pain defined as FLACC ≥4. For the description of the best-fit cut-off values, Youden's point (highest combined sensitivity and specificity) was used.

## Results

### Patient characteristics

We initially recruited 80 patients who met the inclusion criteria during the study period. Eight patients were excluded due to protocol violation and technique problem. Finally, data from 72 patients were included for final analysis. The flow chart of the study is present in Figure 1. Clinical characteristics of these patients are shown in Table 1. The patients were 62 boys and 10 girls with an average age of 4.2 ± 1.5 years and an average weight of 18.5 ± 4.6 kg.

**Table 1:** Patient clinical characteristics.

Patient characteristics (*n* = 72)	Values
Age (years)	4.2 ± 1.5
Gender (male/female)	62/10
Height (cm)	106.7 ± 12.5
Weight (kg)	18.5 ± 4.6
BMI (kg/m^2^)	16.1 ± 1.8
ASA (I/II)	50/22
Intraoperative opioids amounts	2 μg/kg (fentanyl)

BMI, body mass index; ASA, American Society of Anaesthesiologists.

Data are expressed as mean ± SD.

### Predictive value of SPI, PTI, HR, and MAP for postoperative acute pain

**Figure 2:**
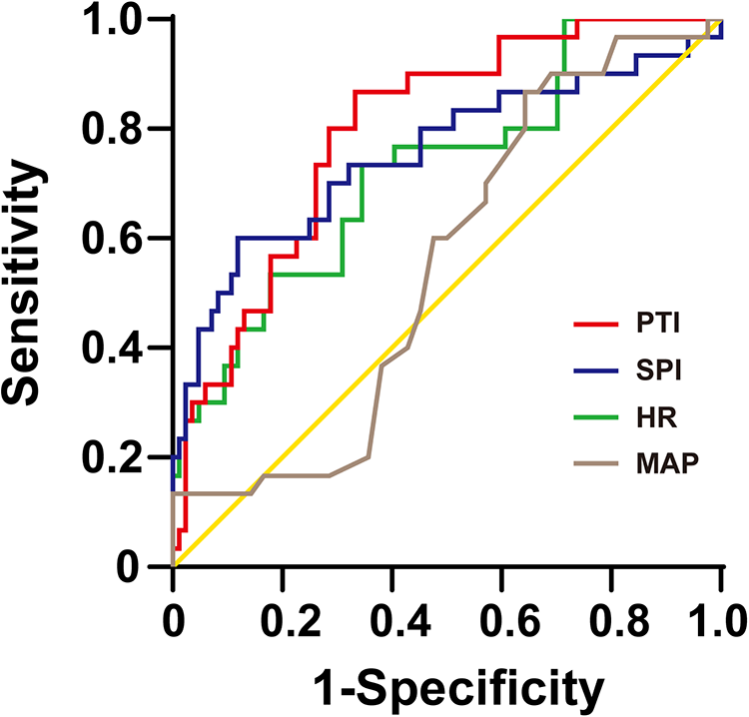
The ROC curves of PTI, SPI, HR, and MAP to distinguish between mild (0–3) and moderate-to-severe (4–10) postoperative pain (FLACC, 0–10). ROC, receiver-operating characteristic; SPI, surgical pleth index; PTI, pain threshold index; HR, heart rate; MAP, mean arterial pressure.

To analyze the ability of the SPI, PTI, HR and MAP to predict postoperative acute pain, ROC curves were plotted and the associated AUCs were calculated. The results showed that PTI (AUC: 0.796, 95% CI: 0.694–0.895, *P *< 0.05) had a good predictive accuracy for postoperative acute pain compared with SPI (AUC: 0.753, 95% CI: 0.63–0.87, *P *< 0.05), HR (AUC: 0.724, 95% CI: 0.61–0.84, *P *< 0.05) and MAP (AUC: 0.551, 95% CI: 0.42–0.69, *P *> 0.05). According to ROCs, the best-fit (highest combined sensitivity and specificity) cut-off values were defined to distinguish mild pain from moderate-to-severe pain after surgery. The cut-off value of PTI were 58 with the sensitivity of 0.80 and specificity of 0.71. In contrast, the cut-off values of SPI and HR were 45 with sensitivity of 0.60 and specificity of 0.88 and 87 with sensitivity of 0.73 and specificity of 0.64, respectively. The ROC curves of these indexes to predict postoperative acute pain are displayed in Figure 2.

We calculated the negative predictive values (NPVs) of PTI and SPI and found that the PTI < 58 had an 80.6% (95% CI: 67.7%–93.5%) NPV to exclude moderate to severe pain. The NPV for SPI < 45 for the absence of moderate to severe pain was 73.5% (95% CI: 61.1%–85.9%).

### Consistency between SPI and PTI at the end of surgery

**Figure 3:**
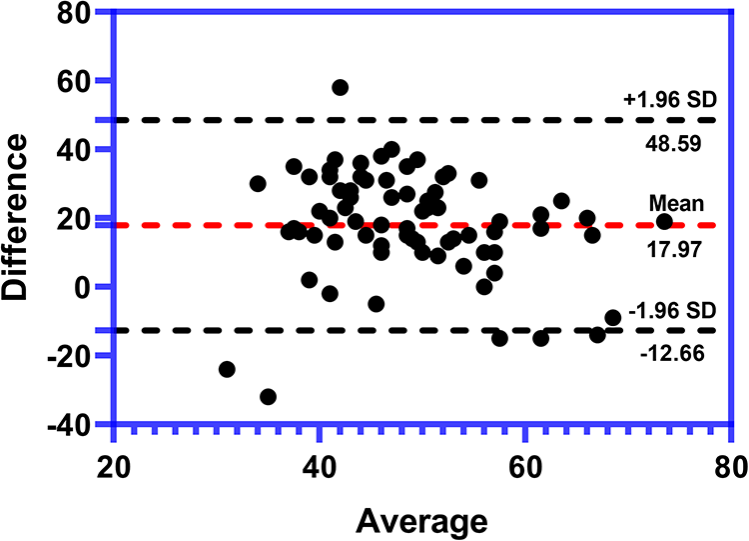
Bland–Altman plot of the consistence between PTI and SPI at the end of surgery (*n* = 72). The average difference (bias) between PTI and SPI is 17.97. The SD of the difference is 15.62. There is a 4.1% (3/72) of all plots outside the agreement lines suggesting a good consistency between PTI and SPI. SPI, surgical pleth index; PTI, pain threshold index; SD, standard deviation.

The values of SPI and PTI were measured simultaneously at the end of the surgery. To study whether PTI had good agreement with SPI, the Bland–Altman plot was used. The Bland–Altman plot of the difference between PTI and SPI against the mean of PTI and SPI in the 72 patients showed that the mean difference (bias) of the measurements between PTI and SPI is 17.97. The SD of the difference is 15.62. The 95% limit contains 95% (69/72) of the difference values, which revealed that there is a good consistency between PTI and SPI, suggesting that PTI can be used to measure pediatric pain nociception under general anesthesia like SPI. The result was shown as Figure 3.

## Discussion

In this study, we found that PTI obtained at the end of the surgery could predict the occurrence of acute postoperative pain with an acceptable accuracy in children. Furthermore, there was a good consistency between PTI and SPI at the end of pediatric surgery, which is in line with adult patients. In addition, HR instead of MAP can also be used as an indicator for acute postoperative pain.

PTI as a novel EEG-derived pain recognition index has been used to predict hemodynamic reactivity induced by trachea intubation and skin incision in pediatric patients and predict acute postoperative pain in adult patients between mild and moderate-to-severe pain (8). In this study, we further confirmed that PTI obtained at the end of surgery could also predict the occurrence of acute postoperative pain and distinguish moderate-to-severe from mild postoperative pain in 2–7 year old children. The area under the ROC was 0.796, and the best cut-off value of PTI was 58, which is consistent with previous adult studies.

It is well-known that SPI derived from the analysis of the plethysmographic pulse wave amplitude and interval could reflect the different levels of autonomous nerve system activation, which is often caused by pain stimulation. Mounting studies have shown that SPI has the potential to reflect intraoperative noxious stimulation and guide the administration of opioids in adult patients (10, 11), and the cut-off value of 50 was used to distinguish between mild and moderate-to-severe pain (12). However, different from adult patients, the study by Ledowski et al. showed that the cut-off value of SPI 50 may be too high to predict acute postoperative pain in children, and its use in children may cause more postoperative agitation and fentanyl consumption (13). They found that the “best-fit” cut-off value of SPI 40 in children aged 2–16 years old was more useful to distinguish between mild and moderate-to-severe pain with high NPV. Furthermore, a large interindividual difference existed among children, which was strongly influenced by age (14). In our study, we limited the children's age to 2–7 years and avoided the use of drugs that affect the sympathetic nerve tone, such as atropine, ketamine, dexmedetomidine, opioids, etc., and found that SPI was able to predict postoperative acute pain with the AUC of 0.753 and the cut-off value of 45, which was similar to the results published recently (40). The NPV for SPI <45 for the absence of moderate to severe pain was 73.5%. Therefore, our study further validated that SPI could be used to predict postoperative acute pain in children with a relatively lower cut-off value compared to adult patients.

Although our study also demonstrated that the PTI has a good consistency with SPI in predicting the incidence of acute postoperative acute pain in pediatric patients, SPI is affected by heart rates or rhythm changes induced by drugs affecting sympathetic tone or changing HR, cardiac diseases, and changes in blood volume. Therefore, these factors should be ruled out when SPI is used to reflect the noxious stimulation, which is quite different from PTI since it is based on EEG wavelet analysis.

The changes of hemodynamic parameters including HR and blood pressure are usually used to monitor the response to noxious stimulations and reflect whether the analgesic level is sufficient during general anesthesia. In our study, we found that HR instead of MAP can also be used as an indicator for acute postoperative pain. However, the heart rate changes rapidly and is easily affected by external factors. Also, in children, heart rate is highly variable with age, so we cannot yet conclude that heart rate can predict postoperative pain, and further research is needed.

There are also some limitations in our study. First, only children aged 2–7 years old were included into our study, and our result cannot be extended to patients younger than 2 years or older than 7 years. Second, when PTI was measured at the end of surgery, the WLI value reflecting the sedative depth was restricted to less than 60, whether WLI value over 60 can affect the predictive accuracy of PTI is unknown. Third, no vasoactive drugs and opioid antagonists were used in our study. Whether these medicines can affect the predictive accuracy of PTI is also unknown. In addition, due to the confidential consideration, we cannot provide more detailed explanation about the algorithm used by this equipment. The possible mechanism we can speculate is that PTI reflects the cortical pain threshold state before noxious stimulations through separating the integrated scalp EEG signals into cortical and subcortical neuronal activities using EEG wavelet analysis. More studies are needed to confirm this approach.

In conclusion, PTI obtained at the end of the surgery could be used to predict the incidence of acute postoperative pain in children aged 2–7 years with an acceptable accuracy and it has a good consistency with SPI. The index can help with early recognition and treatment of postoperative pain, thus improving perioperative comfort and satisfaction of children.

## Data Availability

The original contributions presented in the study are included in the article/Supplementary Material, further inquiries can be directed to the corresponding author.
